# Evaluating the Effectiveness and Cost-Effectiveness of Seizure Dogs in Persons With Medically Refractory Epilepsy in the Netherlands: Study Protocol for a Stepped Wedge Randomized Controlled Trial (EPISODE)

**DOI:** 10.3389/fneur.2020.00003

**Published:** 2020-01-22

**Authors:** Valérie Wester, Saskia de Groot, Tim Kanters, Louis Wagner, Jacqueline Ardesch, Isaac Corro Ramos, Marie-Jose Enders-Slegers, Martine de Ruiter, Saskia le Cessie, Jeanine Los, Grigorios Papageorgiou, Job van Exel, Matthijs Versteegh

**Affiliations:** ^1^Erasmus School of Health Policy & Management, Erasmus University Rotterdam, Rotterdam, Netherlands; ^2^Institute for Medical Technology Assessment, Erasmus University Rotterdam, Rotterdam, Netherlands; ^3^Kempenhaeghe & MUMC+, Academic Centre for Epileptology, Heeze, Netherlands; ^4^Stichting Epilepsie Instellingen Nederland, Zwolle, Netherlands; ^5^Faculty of Psychology, Open University, Heerlen, Netherlands; ^6^Institute for Anthrozoology, Ammerzoden, Netherlands; ^7^Department of Clinical Epidemiology and Department of Biomedical Data Sciences, Leiden University Medical Centre, Leiden, Netherlands; ^8^Department of Biostatistics, Erasmus University Medical Centre, Rotterdam, Netherlands

**Keywords:** seizure dog, refractory epilepsy, stepped wedge randomized controlled trial, economic evaluation, study protocol

## Abstract

**Background:** Epilepsy is associated with a high disease burden, impacting the lives of people with epilepsy and their caregivers and family. Persons with medically refractory epilepsy experience the greatest burden, suffering from profound physical, psychological, and social consequences. Anecdotal evidence suggests these persons may benefit from a seizure dog. As the training of a seizure dog is a substantial investment, their accessibility is limited in the absence of collective reimbursement as is seen in the Netherlands. Despite sustained interest in seizure dogs, scientific knowledge on their benefits and costs remains scarce. To substantiate reimbursement decisions stronger evidence is required. The EPISODE study aims to provide this evidence by evaluating the effectiveness and cost-effectiveness of seizure dogs in adults with medically refractory epilepsy.

**Methods:** The study is designed as a stepped wedge randomized controlled trial that compares the use of seizure dogs in addition to usual care, with usual care alone. The study includes adults with epilepsy for whom current treatment options failed to achieve seizure freedom. Seizure frequency of participants should be at least two seizures per week, and the seizures should be associated with a high risk of injury or dysfunction. During the 3 year follow-up period, participants receive a seizure dog in a randomized order. Outcome measures are taken at multiple time points both before and after receiving the seizure dog. Seizure frequency is the primary outcome of the study and will be recorded continuously using a seizure diary. Questionnaires measuring seizure severity, quality of life, well-being, resource use, productivity, social participation, and caregiver burden will be completed at baseline and every 3 months thereafter. The study is designed to include a minimum of 25 participants.

**Discussion:** This protocol describes the first randomized controlled trial on seizure dogs. The study will provide comprehensive data on the effectiveness and cost-effectiveness of seizure dogs in adults with medically refractory epilepsy. Broader benefits of seizure dogs for persons with epilepsy and their caregivers are taken into account, as well as the welfare of the dogs. The findings of the study can be used to inform decision-makers on the reimbursement of seizure dogs.

## Introduction

Epilepsy is a common neurological disorder, affecting ~50 million people worldwide (1, 2). The disorder is characterized by recurrent unprovoked seizures which, for most persons, can be controlled with antiepileptic drug (AED) regimens. In about one third of the persons with epilepsy (PWE), seizure freedom is not achieved despite the use of multiple AED regimens at therapeutic dosage (3). These persons are considered to have medically refractory epilepsy (4). Resective surgery may be a treatment option for persons with medically refractory epilepsy. However, only about half of PWE meets the strict eligibility criteria, and about 50% of procedures does not result in sustained seizure freedom (5, 6). Neurostimulation has offered new treatment options in medically refractory epilepsy with vagus nerve stimulation and deep brain stimulation, however, these advances seldomly result in seizure freedom (7, 8). Consequently, a group of persons continues to face the challenges of living with persistent seizures.

### Burden of Medically Refractory Epilepsy

Seizures are associated with several risks, leading to increased rates of mortality (9–12) and morbidity (13, 14) in PWE. Causes for excess mortality include status epilepticus and seizure-related accidents (12, 15, 16). However, the most important contributor of excess mortality is sudden unexpected death in epilepsy (SUDEP) (17–20), that is, death occurring without finding a toxicological or anatomical cause. While rare among persons with new-onset epilepsy, seizure-related deaths may account for up to 40% of all deaths in persons with medically refractory epilepsy (15, 21, 22). PWE on average have a lower quality of life, for instance due to medication side-effects and seizure-related injuries. Psychological distress is also a common concern in epilepsy, with PWE being twice as likely to report depression and anxiety compared to the general population (23, 24). While persons with medically refractory epilepsy represent the minority of PWE, they account for most of the disease burden of epilepsy (25).

In addition to the health impact, medically refractory epilepsy comes with restrictions in several life domains including housing, education, occupation, transportation, and role expectations. As a consequence, PWE might feel restricted in their social life and independence as they generally rely heavily on informal care or community services. Finally, it is widely acknowledged that refractory epilepsy can be considered a cost-intensive disorder, with estimates of the indirect costs (productivity and informal care-related costs) generally exceeding the direct costs (treatment-related costs) (26, 27). As such, medically refractory epilepsy imposes a burden not only on PWE, but also on their families and on society as a whole (28, 29).

### Seizure Dogs

The seemingly unpredictable nature of seizures is often considered to be the most disabling aspect of the disorder. Timely detection therefore is essential and can reduce the risks that accompany seizures through early intervention. This has caused PWE and researchers to seek out tools that can help with detecting seizures and/or alarming caregivers during seizures. Technological solutions so far have not been able to recognize all types of seizures (30), which may explain the sustained interest in seizure dogs. Seizure dogs can be trained to detect a variety of seizure types, as they are aware of changes in body movements as well as physiological signals. Furthermore, seizure dogs are able to interact with the PWE in an active way and can recognize seizures based on the observed level of awareness. An additional benefit is that seizure dogs are trained to act upon a seizure by showing a specific response during or immediately after the seizure. This way, seizure dogs may enable timely intervention which can help reduce subsequent seizures and physical risks. Exploratory studies on seizure dogs have shown a reduction in seizure frequency and improvements in quality of life of the PWE (31–36), indicating that persons with medically refractory epilepsy benefit from the companionship of a seizure dog.

While patient organizations and media have shown a sustained interest in seizure dogs over the last decade, the topic remains poorly investigated. Previous studies assessing seizure dogs were mainly retrospective, anecdotal reports with the risk of substantial reporting bias (31–34, 36). The definition of a seizure dog varied across these studies, with some studies including untrained (pet) dogs. Further limitations were limited or no verification of the epilepsy diagnosis and lacking control groups. The only prospective study was conducted by Strong et al. (35), following 10 PWE in a non-randomized design. While the study suggests seizure dogs may reduce seizure frequency, no prospective studies substantiating this expectation have been published. Yet, recent reviews acknowledge the need for further evidence to support the effectiveness of seizure dogs (37, 38).

### Political Rationale for a Study on Seizure Dogs in the Netherlands

One of the consequences of the scarce evidence in this field is that seizure dogs are not reimbursed in most countries. To substantiate reimbursement decisions, decision-makers require evidence of effectiveness and safety, with randomized controlled trials generally being the preferred study design. Additional evidence requirements may apply, such as cost-effectiveness data being a prerequisite for instance in The Netherlands and in The United Kingdom. Without collective reimbursement the accessibility of seizure dogs is limited, as is seen in The Netherlands where PWE generally rely on out-of-pocket payment, donations, or crowdfunding. Affordability issues alongside encouraging anecdotal reports of PWE who privately financed a seizure dog have brought the reimbursement issue to the attention of the Dutch healthcare authorities. As the current evidence base was found to be insufficient to support a positive reimbursement decision (39), the EPISODE (EPIlepsy SuppOrt Dog Evaluation) study was commissioned to inform decision-makers on the reimbursement of seizure dogs in the Netherlands (40).

This protocol describes the EPISODE study which aims to evaluate the effectiveness and cost-effectiveness of seizure dogs combined with usual care in comparison with usual care only in adults with medically refractory epilepsy.

## Methods and Analysis

### Study Design

The study is designed as a prospective stepped wedge randomized controlled trial (41, 42), where randomization occurs at the individual level. In this design, all participants begin in the control phase and sequentially move to the intervention phase in a randomized order. This design was chosen because it allows for rollout of the intervention to all participants. In the case of a standard randomized controlled trial the risk of drop-out during the 3 year follow-up was anticipated to be substantial among those randomized to the control group, especially as blinding of the participants would be impossible. An additional advantage of the stepped wedge design is that it allows for staged implementation, which was inevitable in the context of the current study because the intervention involves intensive selection and training of dogs. The current capacity of the assistance dog schools participating in the EPISODE study would not permit simultaneous rollout of the required number of seizure dogs to all participants.

Data collection started in June 2019 and will take 3 years to complete. To evaluate the effectiveness and cost-effectiveness of seizure dogs, all outcome measures will be taken at multiple time points both before and after the allocation of participants to the intervention.

### Participants

The study population consists of adult persons with medically refractory epilepsy who experience persistent seizures despite currently available treatment options. The seizures should be associated with a high risk of injury or dysfunction. In addition, the welfare of the dog has to be ensured. Inclusion and exclusion criteria are presented in Table 1.

**Table 1:** Inclusion criteria.

**Inclusion criteria**	**Exclusion criteria**
• Aged 18 years or older; • Confirmed diagnosis of epilepsy by a neurologist with video or electroencephalographic (EEG) confirmation; • Failure of adequate trials of two tolerated, appropriately chosen and used AED regimen to achieve sustained seizure freedom (i.e., medically refractory epilepsy); • Not eligible for resective surgery, not prepared to accept the risks of resective surgery, or resective surgery has not resulted in seizure freedom; • Prepared not to commence with a ketogenic diet during the study period; • Minimum of 2 seizures per week on average over the last 6 months^a^; • High risk of injury or dysfunction due to seizures associated with reduced awareness and/or a loss of balance; • Seizures are not preceded by a warning signal that allows the PWE to act on the impending seizure; • Capacity to take care of the dog and back-up support in the environment of the PWE to temporarily take over the care of the dog if necessary; • Adequacy to fill in questionnaires and seizure diaries, either alone or with the support of a caregiver.	• Predominance of psychogenic non-epileptic seizures; • Institutionalized in a 24/7 care home; • Currently in possession of a trained (either active or retired) seizure dog; • A planned epilepsy surgery, deep brain simulation surgery, or vagus nerve stimulation surgery within the trial duration; • Disabilities that would require additional, non-seizure related assistance dog tasks (e.g., dependence on a wheelchair).

a *All types of epileptic seizures may be included in the seizure count. However, given the further criteria (e.g., high risk of injury and/or dysfunction), PWE with tonic-clonic seizures, tonic seizures, clonic seizures, atonic seizures, and/or some focal dyscognitive seizures are most likely to be included in the study*.

### Recruitment and Inclusion

Clinical experts at the academic center for epileptology (Kempenhaeghe) and the expertise center for epilepsy and sleep medicine (SEIN) will inform PWE fulfilling the inclusion criteria about the study. In addition, the assistance dog schools, the Dutch Epilepsy Foundation, and the Dutch Epilepsy Association will bring the study to the attention of PWE by promoting the study through their communication channels (e.g., newsletter, social media, events).

The inclusion process consists of three phases. First, the PWE gives informed consent and hands in an eligibility statement from their treating neurologist. Subsequently, a neurologist from the study team double-checks eligibility based on the medical file and reaches out to the treating neurologist when needed. Finally, the assistance dog school pays a visit to the person's home to assess the suitability of the living situation and the support network of the PWE.

### Intervention

Seizure dogs are trained to detect seizures and to respond during or immediately after a seizure. Different seizure types require different responses. Responses include, but are not limited to: summoning help by the activation of an alarm system or warning someone, helping the PWE to a safe place or position during or after a seizure, blocking the PWE during episodes of reduced awareness from walking into obstacles or dangerous areas (e.g., crossing the street), and providing comfort/emotional support to the PWE until the seizure subsides. The specific set of tasks is tailored to the individual, taking into account the PWE's seizure characteristics, capabilities, support network and living environment. There are reports of dogs that allegedly anticipate on impending seizures and provide a warning signal. However, research to confirm the innate seizure-alerting abilities of dogs has been inconclusive and the presence of this behavior cannot be guaranteed or trained by assistance dog schools.

As the training of seizure dogs is time intensive, two assistance dog schools have been selected to deliver the training program for the seizure dogs that participate in the study: Hulphond Nederland (HN) and Bultersmekke Assistancedogs (BMA). Participants will receive a seizure dog from the school of their preference, striving for an equal balance between the schools. Both schools adhere to the standards of Assistance Dogs International for the training of seizure dogs and use the same endpoints for qualification. The schools, however, use different training protocols. At HN, the dog is trained in a host family and in a kennel before moving in with the PWE at an age of ~20 months. The training of BMA takes place entirely at the PWE's home, starting when the dog is ~8 weeks old. In both schools the training of a seizure dog takes around 24 months to complete. The training consists of ~1 year of basic assistance dog training (i.e., simple commands and socialization) followed by ~1 year of epilepsy-specific training, whereby the focus is on recognizing and responding to seizures. The detailed training schedules of both assistance dog schools can be found in Supplement 1. Participants will continue to receive care as usual during the intervention phase next to having the seizure dog. Participants can keep their seizure dog after the study has ended.

### Comparator

Participants in the control phase will receive usual care without a seizure dog. Usual care consists of AED treatment, which may be complemented by vagus nerve stimulation and/or deep brain stimulation. Some participants might use detection and alarm devices in addition to their treatment. These devices are not commonly reimbursed in the Netherlands, but can be purchased privately.

### Best Medical Practice

Participants will continue to get best medical practice for the entire study duration. Hence, changes in AED regimen or in the settings of the vagus nerve stimulator or the deep brain stimulator are allowed. Treatment details and the use of detection and alarm devices will be recorded to be able to adjust for these changes in the analysis.

### Outcomes

#### Primary Outcome Measure

**Figure 1:**
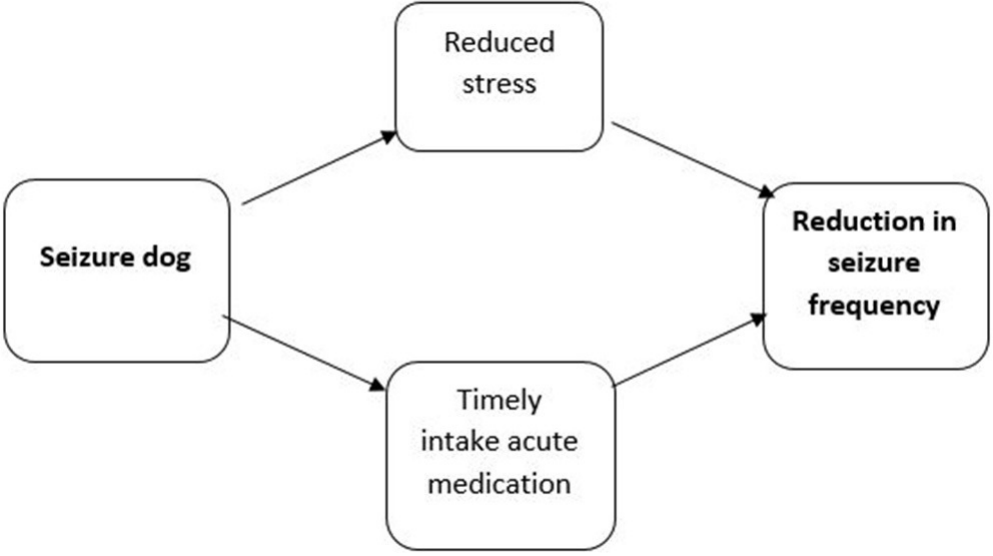
Theorized relationship between the response function of a seizure dog and seizure frequency.

The primary outcome of this study is *seizure frequency*, measured per 28 days. The frequency of seizures is an important indicator for the severity of epilepsy and is therefore one of the most widely used outcomes in the literature on seizure dogs (31–36). The knowledge that the dog will be present and is able to respond during a seizure can be reassuring for PWE, which may reduce seizure worry and stress. As stress is known to be an important trigger for seizures (43, 44), the response function of the dog is expected to result in a decreased seizure frequency. Moreover, as the dog will be trained to warn someone, this may result in administering rescue medication on time and decreasing the likelihood of sequential seizures. Hence, it is theorized that the companionship of a seizure dog will reduce seizure frequency. As the mechanism through which the seizure dog reduces seizure frequency is unknown, we work with a simplified causal model (Figure 1).

#### Secondary Outcome Measures

In addition to the effect of seizure dogs on seizure frequency, the seizure dog might be beneficial for a broader range of outcomes. Focusing only on seizure frequency might therefore underestimate the full effect of seizure dogs, especially since not much is known about the mechanisms by which seizure dogs might impact the PWE's health. Therefore, additional measures of health outcomes (such as quality of life) will be administered through questionnaires. Furthermore, the seizure dog might prove beneficial to PWE beyond their health. To capture the broader individual and societal benefits of seizure dogs, data will be collected on well-being, productivity, resource use, and social participation (38). Finally, as the seizure dog may have an impact on the burden of providing informal care to PWE, the PWE's primary caregiver will be invited to fill in questionnaires as well. Table 2 provides an overview of all the outcome measures that will be administered during the study. All questionnaires will be administered in Dutch.

**Table 2:** Overview of outcome measures.

**Domain**	**Instrument used**	**Measured in participant**	**Measured in primary caregiver**
**BACKGROUND AND DESCRIPTIVES**			
Demographic information (e.g., age, gender, education, living situation).	Structured questionnaire	✓	✓
Disease and treatment characteristics (e.g., seizure characteristics, age at diagnosis, epilepsy treatment history, current epilepsy treatment, use of detection devices, comorbidities).	Structured questionnaire	✓	
**HEALTH**			
Seizure frequency (per seizure type).	Seizure diary	✓	
Seizure-related injuries (categorized as light, mild or severe, and use of rescue medication/magnets to achieve seizure cessation).	Seizure diary	✓	
Seizure severity in terms of (post)ictal phenomena, postictal duration, automatisms, functional impairment, injuries, and warnings.	Dutch translation and adaptation of the NHS-3 (45)	✓	
Epilepsy-specific quality of life in terms of seizure worry, emotional well-being, energy/fatigue, cognition, medication side-effects, social function, and overall quality of life.	QOLIE-31-P (46)	✓	
Generic quality of life in terms of mobility, self-care, usual activities, pain/discomfort, anxiety/depression, and overall quality of life.	EQ-5D-5L and EQ-VAS (47)	✓	✓
**WELL-BEING**			
Well-being in terms of attachment, stability, achievement, enjoyment, and autonomy.	ICECAP-A (48)	✓	
**RESOURCE USE**			
Utilization of healthcare in terms of events (e.g., emergency department visits, ambulance calls, hospitalizations) and total healthcare costs including informal care.	iMCQ (49)	✓	
**PRODUCTIVITY**			
Employment, absenteeism (days missed from work), presenteeism (reduced productivity at work), and unpaid work.	iPCQ (50)	✓	
**SOCIAL PARTICIPATION**			
Participation in household activities, social contact, role expectations, societal participation, and leisure activities.	Structured questionnaire	✓	✓
**CAREGIVER BURDEN**			
Objective burden (e.g., duration and intensity), subjective burden (e.g., perceptions of strain), and health and well-being effects of providing informal care.	iVICQ (51)		✓
**DOG'S RESPONDING/ALERTING BEHAVIOR**			
Performance of response tasks during or after the seizure (e.g., activation of alarm system, bringing medication, blocking the PWE) and observations of alerting behavior (e.g., warn prior to seizure).	Seizure diary	✓	

*NHS-3, National Hospital Seizure Severity Scale; QOLIE-31-P, Patient-Weighted Quality of Life in Epilepsy Inventory-31; EQ-5D-5L, EuroQol 5-Dimensions 5-Levels Questionnaire; EQ-VAS, Euroqol Visual Analoque Scale; ICECAP-A, ICEpop CAPability measure for Adults; iMCQ, iMTA Medical Consumption Questionnaire; iPCQ, iMTA Productivity Costs Questionnaire; iVICQ, iMTA Valuation of Informal Care Questionnaire*.

### Randomization Procedure

**Figure 2:**
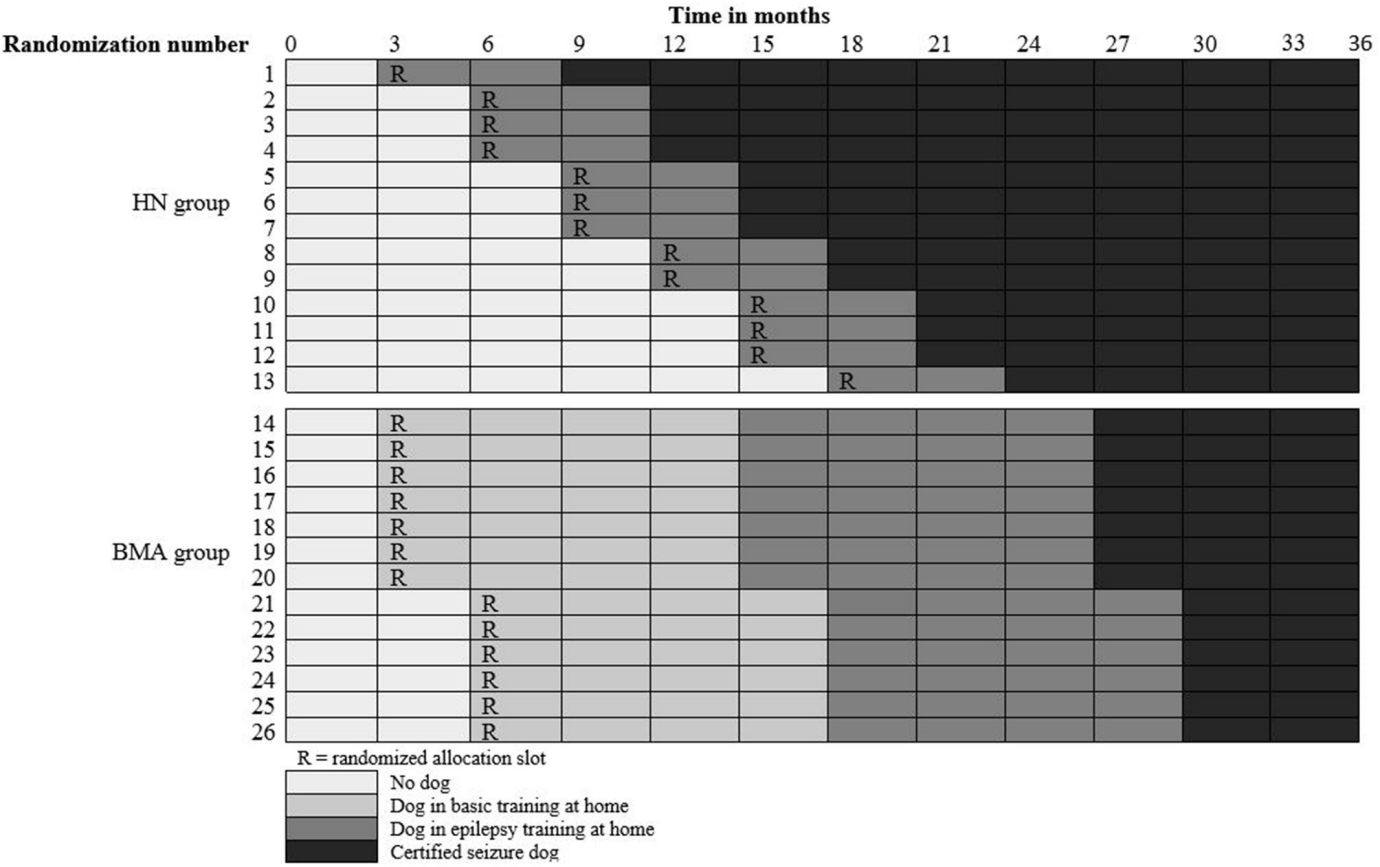
Stepped wedge schedule.

Due to the difference in training protocols, participants can only be randomized within their assistance dog school group (i.e., BMA or HN). Hence, stratified randomization will be applied. In the BMA group, the randomization determines the start of the 2 month period in which the participant receives a puppy at home and begins with the basic assistance dog training. The randomization in the HN group determines the start of the 3 month period in which the participant receives a trained dog at home and continues the epilepsy-specific training. The stepped wedge schedule is designed bearing in mind that all participants have a qualified seizure dog before the end of the study, while considering the capacity of the training schools. In addition, the schedule allows for a minimum of three measurements before and three measurements after the intervention for each participant with respect to the primary outcome measure, in line with the Effective Practice and Organization of Care (EPOC) guidelines (52). In the BMA group, this resulted in two allocation slots meaning that multiple participants receive a puppy within the same period. In the HN group, participants were randomized over 13 allocation slots, as trained dogs will become available one at a time. The stepped wedge schedules are shown in Figure 2.

A good match with the PWE is important for the seizure dog to function effectively. Participants in the BMA group will therefore select their own puppy with the guidance of BMA, i.e., a puppy that appears to be a good match with the PWE and seems suitable for assistance dog tasks. In the HN group, participants receive a dog that is available at that moment, i.e., a dog that has finished the basic assistance dog training, is found to be suitable for seizure dog tasks, and appears to be a good match with the PWE. While the randomization determines the order in which participants receive a seizure dog, it might occur that the allocated dog in the HN group does not match with the PWE (e.g., in terms of energy level, living situation, or support network). In that case, the participant switches places with the next participant in the randomized allocation sequence.

### Data Collection

Seizure frequency will be recorded continuously using a paper seizure diary for the duration of the study (36 months). On days when the participant experiences at least one seizure, participants are asked to record seizure-related injuries and the use of rescue medication or a magnet (when the PWE has a vagus nerve stimulator) to achieve seizure cessation. After receiving a puppy (in the BMA group) or dog (in the HN group), participants are also asked to record whether the dog appears to show responding and/or alerting behavior. A mobile phone application will be used to remind participants to fill in their seizure diary. In order to monitor non-response and to limit retrospective entry of seizures, the application will ask participants weekly to upload a photo of their seizure diary. Non-response will be acted on when observed by the daily study coordinator, by contacting the participant through e-mail or phone. The secondary outcome measures will be collected at baseline and every 3 months thereafter, using paper questionnaires.

The cut-off point between control measurements and intervention measurements is determined prior to the analysis, based on the expected ability of the dog to perform seizure-related tasks at the PWEs home. This is described hereafter.

### Sample Size Calculation

The sample size calculation was based on the primary outcome of seizure frequency per 28 days. To determine the required sample size of the study, a simulation-based approach was adopted, using a generalized linear mixed-effects model with subject-specific random-intercepts, including a term for the time on intervention to allow for a change in treatment effect over time. The advantage of a simulation-based approach is that it can consider the specific features of the study including the repeated measurements and the envisioned stepped wedge schedule (53). Estimates of the treatment effect (i.e., time on treatment) were derived from a study by Strong et al. (35). In that study the mean number of seizures in each 4 week period decreased from 13.8 in weeks 1–12 (baseline) to 9.73 in weeks 13–24 (training) to 8.8 in weeks 25–36 (follow-up) and to 8.0 in weeks 37–48 (follow-up). Four different assumptions were used with respect to the timing of the expected decrease in seizure frequency, in line with the analysis plan in Supplement 2. The study power was calculated as the proportion among 2,500 simulation runs that detects the intervention effect at a 5% significance level. The calculation was run for two sample sizes: 20 and 25 participants. The sample size calculation showed that with both sample sizes, the study would detect the effect on seizure frequency per 28 days as observed by Strong et al. (35) regardless of the assumptions with respect to the timing of the expected decrease in seizure frequency (i.e., the power was higher than the pre-specified threshold of 0.8 in all scenarios). The study will be designed to include a minimum of 25 participants to account for drop-outs.

### Analysis

#### Effectiveness Analysis

The effectiveness of seizure dogs will be measured in terms of reduced seizure frequency. All types of epileptic seizures will be included in the main analysis. Data will be described using summary statistics and scatterplots of the time series, in order to identify any underlying trends of seizure frequency, seasonal patterns and outliers.

Generalized linear mixed models (GLMM) or generalized estimated equations (GEE) are deemed as appropriate statistical methods to analyze data from stepped wedge studies (41, 42). As the primary endpoint concerns count data, a GLMM Poisson model with a logarithmic link is deemed most appropriate. The main model will include a term for time on intervention to allow for a gradual change in effect of treatment over time.

The intervention phase consists of two distinct stages: the development stage during which the dog is taught epilepsy-specific tasks at the participant's home, and the period after the dog finished the epilepsy-specific training and is officially certified as “seizure dog.” The cut-off point between the control and intervention period relates to the expected moment at which the dog has the ability to perform seizure-related tasks at the PWEs home. For participants in the HN group, we expect the effect to occur with a delay of 6 months after the dog moves from the kennel to the participant's home to account for the period of acclimatization. For participants in the BMA group we expect a decrease in seizure frequency as soon as the epilepsy-specific training starts, as the acclimatization period already took place during the basic assistance dog training at the participant's home. Hence, for PWE in the BMA group the start of the intervention phase (i.e., time on intervention) is defined as the start of the epilepsy-specific training at the participant's home. More details on the classification of “control” and “intervention” measurements can be found in the analysis plan (Supplement 2).

While seizure clusters are common in refractory epilepsy, there is no agreement on the definition of a cluster. This lack of consensus is revealed in the literature, where definitions that have been referenced range from episodes of multiple seizures within minutes to up to 24 h (54, 55). This study only allows us to group seizure clusters that occur within a given day, as there is no additional granularity in the measurement of time in the seizure diary.

GLMM will be used for the secondary endpoints as well, however, the type of model (Normal, Binomial, Poisson) will be determined by the type of data for each outcome. Conclusions will be drawn from this main analysis on primary and secondary endpoints.

Given the uncertainty surrounding the model assumptions, sensitivity analyses will be conducted to test the implications of the most important choices, such as the starting point of the intervention phase, the inclusion of a main effect, the types of seizures included in the count data and the definition of seizure clusters.

In addition, exploratory analysis will be performed to gain insight into the relationship between the dogs responding and/or alerting behavior and the PWE's health outcomes (e.g., seizure frequency, seizure-related injuries, anxiety).

#### Cost-Effectiveness Analysis

The cost-effectiveness analysis will follow the Dutch guidelines for economic evaluations in healthcare (56). In line with this guideline, the cost-effectiveness analysis will adopt a societal perspective. This implies that the analysis will take into account all costs within the health care sector, patient and family costs (i.e., time costs of informal caregivers and travel costs) and costs in other sectors (i.e., productivity costs). The Dutch costing manual will be used to derive unit costs (57). The intervention costs include the purchase of a puppy, the costs of the training program and follow-up services, and costs for keeping the dog until the dog retires.

The main outcome of the analysis are the incremental costs per quality-adjusted life-year (QALY) gained, expressed as the incremental cost-effectiveness ratio (ICER). The main analysis will use a lifetime time horizon. Lifetime costs and effects will be estimated, assuming that the dogs will be replaced at the time they retire. Future costs and effects will be discounted according to the Dutch guidelines for economic evaluations in healthcare. Scenario analyses will explore the cost-effectiveness of seizure dogs without the assumption that dogs will be replaced when they retire. Additional uncertainties (both structural and parameter) will be tested in scenario- and sensitivity analyses.

### Ensuring the Dog's Welfare

The current animal welfare debate has demonstrated broad acknowledgment that interventions with assistance dogs may raise welfare concerns for participating animals (58, 59). BMA and HN apply internal protocols to monitor the health of seizure dogs and the conditions in which the seizure dogs function, in line with international standards (Assistance Dogs International). Before PWE enter the study, information is provided to the PWE in order to make them aware of the impact of taking care of a dog. Adequacy to ensure the dogs welfare is a criterion for participating in the study. After the dog is placed at the participant's home, the welfare of the dog will be monitored regularly by the assistance dogs schools. The Institute for Anthrozoology will conduct a side-study to evaluate the welfare of the seizure dogs involved in the current study.

## Discussion

This study is the first randomized controlled trial concerning the effectiveness and cost-effectiveness of seizure dogs. The stepped wedge trial design combined with a 3 year follow-up, and the broad range of outcomes measured, allow for a thorough investigation of the clinical and societal benefits of this intervention. For instance, the study will look beyond health-outcomes, taking into account PWE and caregivers' well-being as well as their participation in social activities. Another neglected outcome in previous studies was the economic benefit of seizure dogs. While the costs of the training program may be considerable, and for some PWE insurmountable when not covered by health insurance, the cost savings due to reduced medical consumption, a reduced informal caregiver burden and increased productivity might compensate this investment. As we will apply a societal perspective, these potential benefits are accounted for in the cost-effectiveness analysis, capturing the full range of expected costs and benefits attributable to seizure dogs to both PWE and society.

### Limitations and Complications

Some limitations in the design of the study deserve mentioning. The study relies on self-reported outcome measures which, particularly in the light of the non-blinded design, have the potential of introducing reporting bias. The incentives to misrepresent responses are expected to be mitigated by several factors. First, participants can keep their dog after the study has ended, independent of the outcome of the study. As a result, the incentive to bias the results in order to increase the chance of keeping the dog will be removed. Second, participants often use the seizure diary of the study also to inform their treating neurologist. Hence, misrepresenting well-being in the seizure diary would result in misinforming ones treating neurologist as well. Third, participants have agreed that the neurologists from the study team may access their medical record for validation purposes. Finally, the analyses plan differentiates between the intervention period and the training period, and participants are neither aware of these differences nor can forecast the impact of changing responses in different phases of the study, limiting the impact of strategic responses. Nevertheless, strategic responses may not all be avoided.

Another limitation is that the study design does not allow for a comparison of the benefits of an untrained dog and a trained seizure dog. It has been suggested in the literature that pet ownership at itself may have beneficial effects on their handler's health and well-being (60–63). However, it was not feasible to include a control arm in which participants are assigned to receiving an untrained pet dog. Most importantly, there were concerns for the well-being of the participant and the dog, given the various instinctive ways in which the dog may react to seizures in their human companion when they are not trained which may include aggressive or escape behavior (64). Adding to this, it was expected that very few eligible PWE were willing to enroll in the study when they knew they could be randomized to receiving an untrained pet dog.

A third limitation is that the allocation schedule for the BMA group resulted in only two allocation slots. As a consequence, factors that might influence the results (apart from the intervention) might be less well-balanced over “control” and “intervention.” However, given that the data of the BMA and HN group will be pooled, the impact on the results will be limited.

Some remarks should also be made with respect to the focus of the current study. The current study investigates effectiveness without a full understanding of the mechanism through which seizure dogs reduce seizure frequency as was observed in previous studies. However, the political relevance of the effectiveness and cost-effectiveness question is believed to surpass the uncertainty regarding the mode of action and hence it is warranted to measure effectiveness regardless of a clear understanding of how the effect arises. Exploratory analysis (e.g., on the relationship between response/alert behavior and seizure frequency) will be conducted to test hypotheses concerning the mode of action. In addition, it should be noted that the study focuses on seizure dogs that are trained for their response behavior, as training methods for alerting behavior rely on hypothesized cues by dogs (e.g., variations in behavior, scent, heart rate) and cannot be guaranteed in the way that conventional training (i.e., seizure response training) can. Nevertheless, the number of seizures, which will be the primary outcome of the study, might be influenced by both response and alerting behavior. Finally, this study is not designed to evaluate adverse events as data on the type and number of potential adverse events are not registered in a systematic way. However, several processes are in place to optimize the safety of the PWE as well as the dog (e.g., careful selection and thorough training of the dogs as well as regular home visits by the assistance dogs schools), making adverse events seem unlikely. If major concerns regarding patient- or dog safety occur, the dog will be removed from the PWE's home after careful consideration, with or without assigning a replacement dog. These serious adverse events will be recorded by the study team, and described as part of the study results. Aspects of safety that influence a participant's well-being, quality of life or resource use will be measured throughout the study.

## Ethics Statement

The medical ethics committee of the Erasmus Medical Center Rotterdam has reviewed the research proposal and declared that the rules of the Medical Research Involving Human Subjects Act (also known by its Dutch abbreviation WMO) do not apply to this study, reference number MEC-2017-538. The medical ethics committee Kempenhaeghe, Heeze, The Netherlands, approves this study. The study is registered with the Dutch Trial Register, part of the Dutch Cochrane Centre (NTR6852). Written informed consent for participation in the study is obtained from all participants prior to the baseline measurement.

## Author Contributions

VW, SG, TK, LW, JA, IC, M-JE-S, MR, SC, JL, JE, and MV contributed to the design of the study. GP performed the sample size calculation. VW was the daily coordinator of the study and drafted the manuscript. All authors commented on the manuscript and approved the final manuscript.

### Conflict of Interest

The authors declare that the research was conducted in the absence of any commercial or financial relationships that could be construed as a potential conflict of interest.

## Abbreviations

AED: Antiepileptic drug
BMA: Bultersmekke assistancedogs
EEG: Electroencephalography
EPISODE: Epilepsy support dog evaluation
EPOC: Effective practice and organization of care
EQ-5D-5L: EuroQol 5 dimensions 5 level questionnaire
EQ-VAS: EuroQol visual analogue scale
GEE: Generalized estimated equations
GLMM: Generalized linear mixed model
HN: Hulphond Nederland
ICECAP-A: ICEpop CAPability measure for Adults
ICER: Incremental cost-effectiveness ratio
iMCQ: iMTA Medical Consumption Questionnaire
iMTA: institute for Medical Technology Assessment
iPCQ: iMTA Productivity Costs Questionnaire
iVICQ: iMTA Valuation of Informal Care Questionnaire
NHS-3: National Hospital Seizure Severity Scale
PWE: Person(s) with epilepsy
QALY: Quality-adjusted-life-year
QOLIE-31-P: Quality of Life in Epilepsy Questionnaire
SEIN: Stichting Epilepsie Instellingen Nederland
SUDEP: Sudden unexpected death in epilepsy.
