# Supplementary material for: Evaluating the Effectiveness and Cost-Effectiveness of Seizure Dogs in Persons With Medically Refractory Epilepsy in the Netherlands: Study Protocol for a Stepped Wedge Randomized Controlled Trial (EPISODE)
Source: Front Neurol. 2020 Jan 22;11:3. doi: 10.3389/fneur.2020.00003 (PMC6987301; doi:10.3389/fneur.2020.00003)

Training programs of participating assistance dog schools

Figure 1: Training of seizure dogs at Hulphond Nederland (HN)

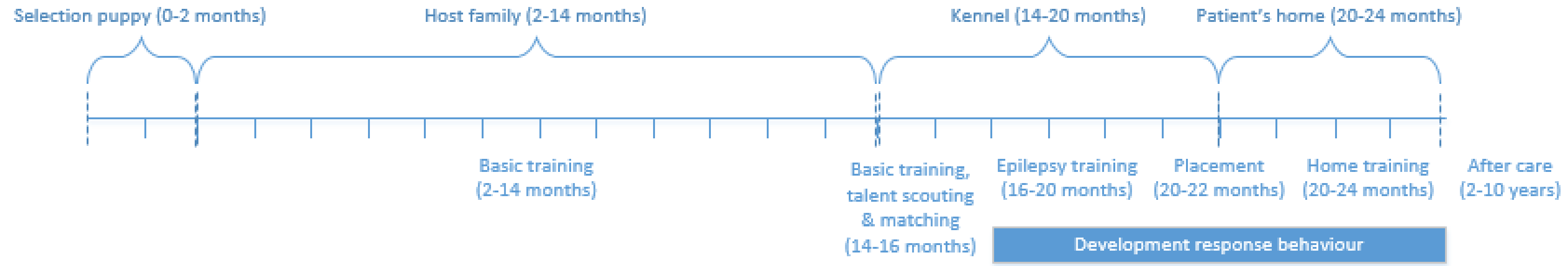

Figure 2: Training of seizure dogs at Bultersmekke Assistancedogs (BMA)

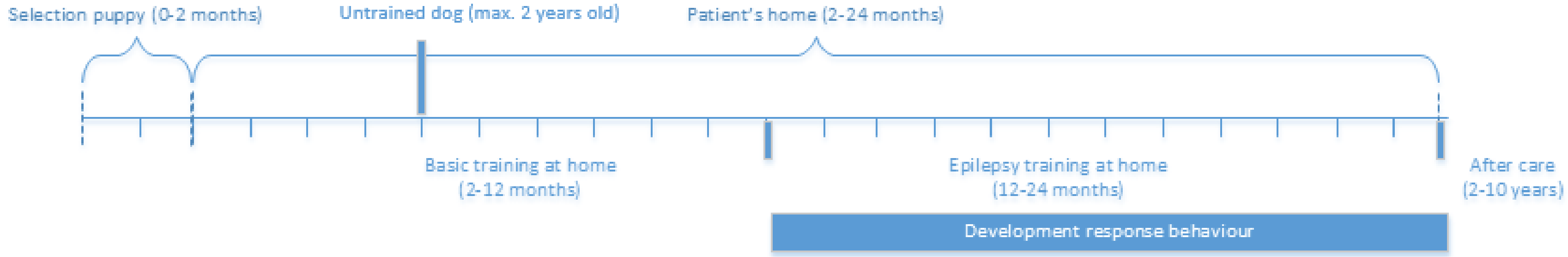

Supplement: Supplementary file 1 [file Data_Sheet_1.PDF]
