# Supplementary material for: Evaluating the Effectiveness and Cost-Effectiveness of Seizure Dogs in Persons With Medically Refractory Epilepsy in the Netherlands: Study Protocol for a Stepped Wedge Randomized Controlled Trial (EPISODE)
Source: Front Neurol. 2020 Jan 22;11:3. doi: 10.3389/fneur.2020.00003 (PMC6987301; doi:10.3389/fneur.2020.00003)
Supplement: Supplementary file 2 [file Data_Sheet_2.PDF]

Statistical Analysis Plan for the (Cost)-Effectiveness Analyses for the EPISODE trial

1. Purpose

The purpose of this statistical analysis plan is to provide the details of the statistical analyses that are planned for the data on clinical effectiveness, cost-effectiveness and broader outcomes (e.g. wellbeing, participation and caregiver burden) of the EPISODE trial.

2. Study design

The EPISODE study is a 3-year stepped-wedge randomized controlled trial [1] [2]that compares the use of seizure dogs with the usual care for adults with refractory epilepsy. In this study, seizure dogs will be introduced sequentially to patients over time in which the order is chosen at random. In other words, patients will progressively move from the control group to the intervention group at random time points. At the end of the trial all patients have received a seizure dog. This staged implementation is inevitable, because simultaneous rollout of trained dogs to all patients is impossible for logistical reasons (i.e. the time and capacity needed to train seizure dogs). The schedule of the staged implementation is designed in such a way that, in line with the Effective Practice and Organisation of Care (EPOC) guidelines on what study designs to include in a systematic review, at least three measurements before and three measurements after the intervention will be planned for each patient [3].

Due to the difference in training protocols, patients will be randomized within their training stratum (i.e. Bultersmekke Assistancedogs or Hulphond Nederland), meaning that randomization is stratified according to training cluster and that there are effectively two randomization procedures. To determine the order in which patients receive the intervention (i.e. the seizure dog) within their stratum, each patient gets assigned a random number between 0 and 1. The patient with the smallest number will be the first one to receive the intervention within the stratum, the patient with the highest number will be the last one.

To evaluate the effectiveness and cost-effectiveness of seizure dogs, all outcome measures (summarized in the next section) are taken at multiple time points both before and after the random allocation to the intervention. This implies that we have for each subject a time series of measurements for each of the outcomes of interest. This time series of measurements will be used to establish an underlying trend, which is ‘interrupted’ by the intervention at a known (and

randomly assigned) point in time [4]. In this way, we may detect whether the intervention has had an effect significantly greater than any underlying trend over time [5].

3. Study endpoints

Primary outcome measure

1. Seizure frequency over 28 days

Seizures are recorded continuously using a paper seizure diary for 36 months. An app will be used to remind patients to fill in their seizure diary. In order to monitor non-response and to limit retrospective entry of seizures, the app will routinely ask patients to photograph their seizure diary. Non-response will be actioned upon when observed by the daily study coordinator. Patients will be asked to record all epileptic seizure types. Patients will be asked to record psychogenic non-epileptic seizures as well, but these will be excluded from the main analysis given their non-epileptic nature.

Secondary outcome measures

1. Seizure severity: seizure diary and NHS-3 [6]
2. Generic health-related Quality of Life: EQ-5D-5L [7]
3. Disease-specific health-related Quality of Life: QOLIE-31-P [8]
4. Well-being: ICECAP-A [9]
5. Utilization of healthcare in events (ED visits, ambulance calls, hospitalizations, inpatient days): iMCQ [10]
6. Total healthcare costs (including informal care): iMCQ
7. Productivity losses: iPCQ [11]
8. Social participation: covering the domains social contact, daily activities and leisure activities (patient and caregiver)
9. Caregiver burden: iVICQ [12]

The secondary outcome measures are collected every 3 months using a set of questionnaires. The timing of the three-monthly questionnaire is calculated from the start of the study (t=0), and will be reset after transitioning from control to intervention. These questionnaires will be filled in on paper.

4. (Clinical) assumptions underlying the analysis plan

Primary outcome measure

Seizure frequency is expected to be affected by the introduction of a seizure dog. People with

epilepsy may experience less anxiety due to the companionship of a seizure dog, even when the dog is only responding to seizures [13]. A decrease in seizure worry may reduce stress, which is known as an important trigger for seizures [14]. Reducing stress may be a result of the response function of the seizure dog and may cause a decreased seizure frequency. Moreover, the activation of an alarm button by the dog can inform family or neighbors quickly, resulting in administering the emergency medication on time. Timely medication might reduce seizure frequency due to a decreased likelihood of sequential seizures.

Although it might take some time before the dog has learned how to accurately respond to a seizure, we expect a decrease in seizure frequency shortly after the dog starts epilepsy training (at home), in line with the study by Strong et al. (2002) [15]. Since there is no evidence of any secular trend (i.e. the number of seizures does not decrease with time), or any cyclical or seasonal effects (i.e. there is no cyclical pattern in the number of seizures that occur over time), a slope change leading to a level change in the number of seizures is expected. The slope is assumed to continue until the intervention has reached its full potential (which depends on the time needed for the dog to improve its sensitivity to seizures), after which we expect to observe a plateau (Figure 1).

Figure 1. Expected effect of intervention over time

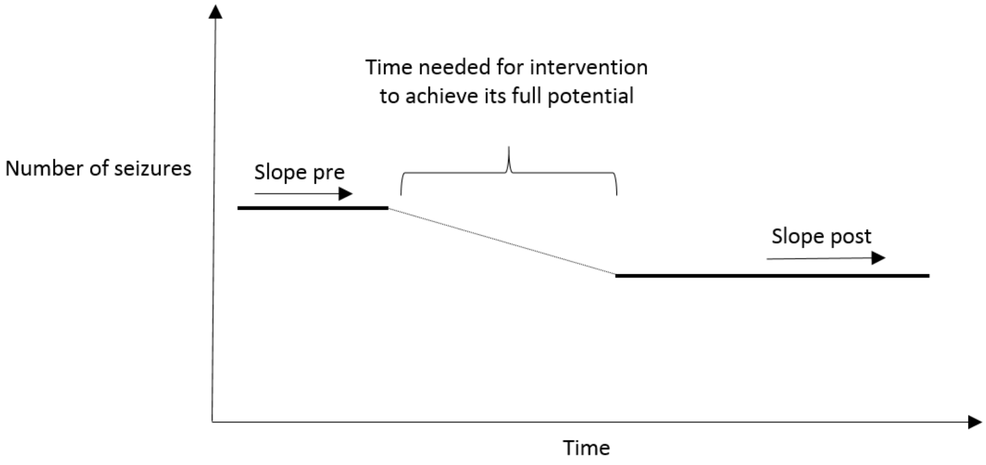

Secondary outcome measures

Besides the effect of seizure dogs on seizure frequency, an effect may be observed on seizure severity. Seizure severity consists of several domains including secondary damage (e.g. cuts, burns or fractures) and the time to recovery, and both may be influenced by the presence of a seizure dog. To elaborate, the dog can get help when the patient has lost consciousness, the dog may block the patient from putting him/herself in danger during impaired consciousness, and the dog's

presence might reduce stress and anxiety during or immediately after the seizure. We, therefore, expect an impact on other outcomes measures as well, such as well-being (e.g. because dogs provide greater independence), health-related quality of life (e.g. due to a reduction of problems with performing usual activities or less feelings of anxiety/ depression), productivity losses and social participation. Furthermore, we expect seizure dogs to reduce healthcare resource use, because seizure dogs might help prevent prolonged hospital stays due to a reduction of secondary damage, such as sequential seizures, or simply because unnecessary ambulance arrivals are avoided. In addition to the effect of seizure dogs on the patients, there might be an impact on their informal caregivers, i.e. patients may need less help of family and friends and, therefore, the burden on informal caregivers may be reduced.

For the secondary outcomes the effect is expected to follow a similar pattern to the effect on seizure frequency. Therefore, outcomes are assumed to be affected shortly after the dog starts epilepsy training (at home) and we expect a temporary slope to appear. Although the causal pathway is unknown, some outcomes may show a delayed treatment effect, for example caregiver burden and participation. These outcomes might not improve immediately, but only after the decreased seizure frequency has been stable for a while. This will be tested in the exploratory analyses.

5. Data analysis

Effectiveness analysis

The effectiveness of seizure dogs will be measured in terms of a change in seizure frequency. Data will be described using summary statistics and scatterplots of the time series in order to identify any underlying trends of seizure frequency, seasonal patterns and outliers [4]. A simple before-and-after comparison will be conducted by calculating per person the average of the measurements before the dog's epilepsy training starts, during the dog's epilepsy training at home and after the training of the dog is completed. We will further describe how often the seizure dog responded to seizures, and how often they detected an oncoming seizure.

<u>Main analysis on primary and secondary end-points</u>

Generalized linear mixed models (GLMM) or generalized estimated equations (GEE) are deemed as appropriate statistical methods to analyze data from stepped wedge studies [16, 17]. The models specified by Hussey and Hughes include time as a fixed effect for each step [17]. Thus, for example, for continuous (and normally distributed) outcomes, a model with random effect for cluster and fixed effect for each step (time) is suggested. Note that in our case the size of the cluster

is 1 patient. Likewise, for binary outcomes a logistic regression model is recommended and for count outcomes a Poisson regression model is appropriate.

Primary end-point

For the primary end-point we are dealing with count data (number of seizures over 28 days ranging from 0 to n) and since seizure frequency is not normally distributed, a GLMM Poisson model with a logarithmic link will be used. The main model will include a term for the time on treatment, to allow for a gradual increase in treatment effect over time.

We expect a decrease in seizure frequency shortly after the dog starts epilepsy training. Therefore, in the Bultersmekke Assistancedogs group, the time on treatment is defined from the start of the epilepsy training, which commences 12 months after basic training. At Hulphond Nederland the epilepsy training commences 14 months after basic training while the dog is still in the kennel. During this 8 month long epilepsy specific training, the dog is placed at the home of a patient. The dog trainers have experienced that the dog and patient first experience a period of acclimatization during which they bond and the dog needs to get comfortable with the new environment. Therefore they expect that outcomes start improving slightly later due to the disruption in the living environment of the dog: six months after the dog starts living at the home of the patient. In the Bultersmekke Assistancedogs group this acclimatization phase took place during the first year of basic training with the dog. Hence, in the main analysis time on treatment is defined from the start of the epilepsy training in the Bultersmekke Assistancedogs group, and 6 months after the dog has been placed in the home of the patient in the Hulphond Nederland group.

Appendix I shows how predictions from the main model may look like.

Secondary end-points

For the secondary end-points we are dealing with different distributions for the outcomes. The model structure described for the primary outcome will be used for the secondary outcomes (listed under point 3) as well, but the assumptions concerning the distribution of the data will be amended. The type of model (Normal, Binomial, Poisson) will be determined by the type of data for each outcome (continuous outcomes should use Normal, binary outcomes Binomial and count outcomes Poisson).

Conclusions will be drawn from this main analysis on primary and secondary endpoints. The analyses described below are exploratory.

In the exploratory analyses, alternative assumptions will be explored. The exploratory analyses will be performed on both the primary and the secondary end-points (where applicable).

Assumptions with respect to the timing of the expected improvement in seizure frequency

i. The timing of the expected improvement in seizure frequency will be changed. The parameter 'time on treatment' will be defined from the start of the epilepsy training (in the Bultersmekke Assistancedogs group) and from 6 months after the home placement (in the Hulphond Nederland group) in line with the main analysis:
   a. plus two months (2/12 epilepsy training Bultersmekke Assistancedogs; home placement +6 +2 months Hulphond Nederland);
   b. plus four months (4/12 epilepsy training Bultersmekke Assistancedogs; home placement +6 +4 months Hulphond Nederland);
   c. plus six months (6/12 epilepsy training Bultersmekke Assistancedogs; home placement +6 +6 months Hulphond Nederland).

ii. The timing of the expected improvement in seizure frequency will be changed in the Hulphond Nederland group only, and this parameter will be defined from:
   a. home placement;
   b. home placement +2 months;
   c. home placement +4 months.

Assumptions with respect to a main effect besides the effect of time on treatment

iii. A parameter for having a dog in epilepsy training will be added to the main model, to explore whether a main effect is observed in addition to the effect of time on treatment;

iv. A parameter for having a trained dog will be added to the main model, to explore whether a main effect is observed in addition to the effect of time on treatment. A trained dog is defined as a dog that has successfully completed the assistance dog exam;

v. A parameter for having a dog in basic training (i.e. time on treatment [basic training]) will be added to the main model, to explore whether having a dog has an effect even before the dog has learned epilepsy-specific tasks. This parameter will be estimated on data from patients in the Bultersmekke Assistancedogs group only, as they receive a pup that will learn basic tasks in the first year. Dogs of patients in the Hulphond Nederland group stay in a kennel during the first year, so these dogs cannot have any impact on any of the outcomes specified in Section 3.

Assumptions regarding the type of seizures that an epilepsy dog can react on

vi. The dependent variable in the main model will be changed such that it excludes absences and myoclonic jerks;

vii. The dependent variable in the main model will be changed such that it only includes

the worst seizure type in each patient as defined by the baseline NHS-3 scores;

viii. The dependent variable in the main model will be changed such that it includes psychogenic non-epileptic seizures.

Besides testing those assumptions, the following alternative (exploratory) analyses will be run:

i. The main model will be rerun after transforming seizure count to seizure-free days;

ii. The main model will be rerun after changing the parameter ‘time on treatment’ from 0 = not on treatment, 1 = on treatment to fractions between 0 and 1 representing the phase of epilepsy training. E.g. after two months epilepsy training, a patient in the Hulphond Nederland training cluster is assigned a time on treatment fraction of 0.50 (2 out of 4 months completed), whereas a patient in the Bultersmekke Assistancedogs training cluster is assigned a fraction of 0.17 after two months in epilepsy training (2 out of 12 months completed). This analysis allows for two different slopes, as well as for two different starting points for the time on treatment;

iii. Hybrid measurements (i.e. including only those measurements where the dog is not in epilepsy training) will be disregarded and a model will be run including a main effect for having a trained dog only.

<u>Missing and invalid data</u>

An advantage of mixed models, including GLMM, is that they can be used in combination with unbalanced data. Unbalanced data means that the number of measurements and measurement times may vary across patients. Since GLMM can be used in combination with unbalanced data, all patients can be included in the analyses, even if they have missing values or when they left the study prematurely. Since we assume the missing data mechanism to be missing at random or missing completely at random (i.e. not related to either control or intervention), unbiased results will be produced. In the presence of severe missing data, general approaches to this problem (e.g. multiple imputation) will be considered.

This study might also suffer from incorrect data, due to, for example, incorrect data entry (by patients or one of the researchers). In order to evaluate the possible impact of incorrect data, standard statistical techniques will be used to detect potential outliers. In case, it is suspected that the model results will be affected by the presence of outliers, the main model will be run without outliers.

Cost-effectiveness analysis

The cost-effectiveness analysis will follow the Dutch guidelines for economic evaluations in

healthcare [18]. The main outcome of the analysis are the incremental costs per quality-adjusted life-year (QALY) gained, expressed as the incremental cost-effectiveness ratio (ICER). The Dutch tariff will be used to calculate utilities from the EQ-5D-5L scores.

In line with the Dutch guidelines for economic evaluations in healthcare, the cost-effectiveness analysis will adopt a societal perspective. The analysis will take into account all significant health outcomes and costs that result from seizure dogs, regardless of who experiences the outcomes or costs. This means all costs within the health care sector are included as well as patient and family costs (i.e. time costs of informal caregivers and travel costs) and costs in other sectors (i.e. productivity cost). The intervention costs include the costs of the training program of the dog and lifetime costs for keeping the dog. The Dutch costing manual will be used to derive unit costs where possible.

The working life of a seizure dog is eight years. Lifetime costs and effects will be estimated, assuming that the dogs will be replaced at the time they 'retire'. In scenario analyses, the cost-effectiveness of seizure dogs using a lifetime time horizon will be explored, without taking into account that the dogs will be replaced when they 'retire'. We will further explore the cost-effectiveness of seizure dogs using a 8-year time horizon, in line with the working life of one dog. The discount rates will be set at 4.0% for costs and 1.5% for effects as recommended by the Dutch health economic guidelines [18].

6. Implementation

All analyses will be led by Isaac Corro Ramos, statistician working at iMTA. He gave his consent on this analysis plan.

References

[1] C. Brown and R. Lilford, "The stepped wegde trial design: a systematic review," *BMC medical research methodology,* vol. 6, no. 1, p. 54, 2006.

[2] N. Mdege, M. Man, C. Taylor and D. Torgerson, "Systematic review of stepped wedge cluster randomized trials shows that design is particularly used to evaluate interventions during routine implementation," *Journal of clinical epidemiology,* vol. 64, no. 9, pp. 936-948.

[3] (EPOC) EPaOoC, EPOC Resources for review authors: Interrupted time series (ITS) analyses, Oslo: Norwegian Knowledge Centre for the Health Services, 2015.

[4] J. Bernal, S. Cummins and A. Gasparrini, "Interrupted time series regression for the evaluation of public health interventions: a tutorial," *International journal of epidemiology,* no. dyw098, 2016.

[5] B. Reeves, J. Deeks, J. Higgins and G. Wells, "Chapter 13: Including non-randomized studies," *Cochrane handbook for systematic reviews of interventions,* vol. 5, no. 0, 2011.

[6] M. O'Donoghue, J. Duncan and J. Sander, "The National Hospital Seizure Severity Scale: A Further Development of the Chalfont Seizure Severity Scale," *Epilepsia,* vol. 37, no. 6, pp. 563-571, 1996.

[7] EuroQol Group, "EQ-5D-5L," 2010.

[8] JA Cramer, G Van Hammée and N132 Study Group, "Maintenance of improvement in health-related quality of life during long-term treatment with levetiracetam," *Epilepsy behaviour,* vol. 4, no. 2, pp. 118-23, 2003.

[9] H. Al-Janabi and J. Coast, "ICECAP-A," 2010.

[10] C. Bouwmans, L. H.-v. Roijen, M. Koopmanschap, M. Krol, H. Severens and W. Brouwer, "iMTA Medical Cost Questionnaire (iMCQ)," 2013.

[11] C. Bouwmans, M. Krol, H. Severens, M. Koopmanschap, W. Brouwer and L. H.-v. Roijen, "The iMTA Productivity Cost Questionnaire: A Standardized Instrument for Measuring and Valuing Health-Related Productivity Losses.," *Value Health,* vol. 18, no. 6, pp. 753-8, 2015.

[12] R. Hoefmanl, N. V. Exel and W. Brouwer, "iVICQ. iMTA Valuation of Informal Care Questionnaire, Version 1.1," 2013.

[13] L. Wagner, "Seizure dogs," 2011.

[14] A. Ge, E. Gonzalez, S. Lee, Y. Carmenate, M. Collard, T. Dixon-Salazar and e. al, "Seizure triggers in epilepsy patients: a national perspective (S37. 002)," *Neurology,* vol. 88, no. 16

Supplement, 2017.

[15] V. Strong, S. Brown, M. Huyton and H. Coyle, "Effect of trained Seizure Alert Dogs on frequency of tonic-clonic seizures," *Seizure*, vol. 11, no. 6, pp. 402-5, 2002.

[16] K. Hemming, M. Taljaard, J. McKenzie, R. Hooper, A. Copas and J. Thompson, "eporting of stepped wedge cluster randomised trials: extension of the CONSORT 2010 statement with explanation and elaboration," *British Medical Journal*, vol. 363, no. k1614, 2018.

[17] M. Hussey and J. Hughes, "Design and analysis of stepped wedge cluster randomized trials," *Contemp Clin Trials*, vol. 28, pp. 182-91, 2007.

[18] Zorginstituut Nederland, "Guideline for conducting economic evaluations in healthcare [in Dutch: Richtlijn voor het uitvoeren van economische evaluaties in de gezondheidszorg]," 2016.

Appendix I

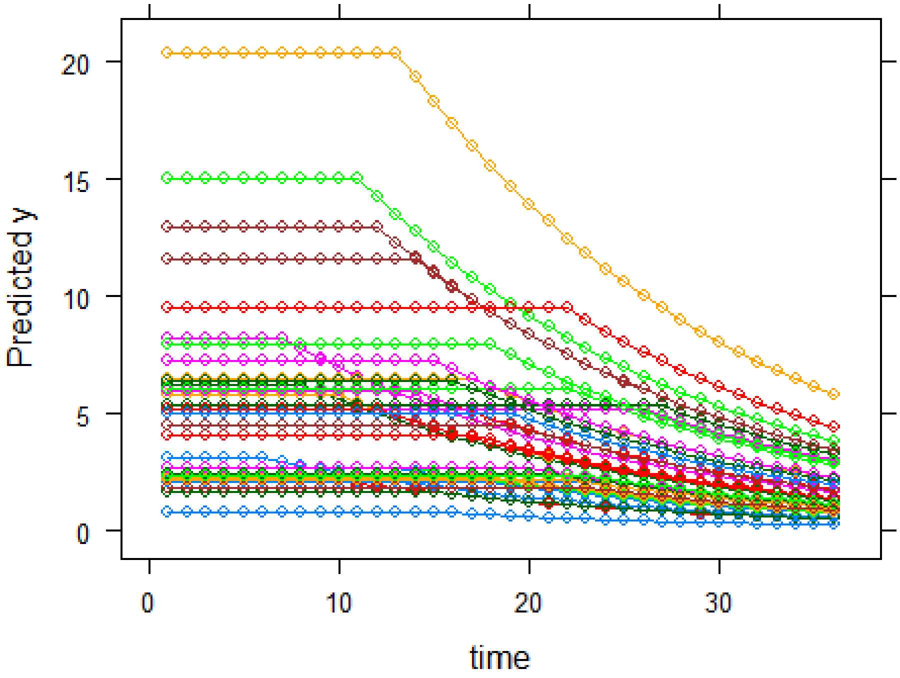

This figure shows how predictions from the main model may look like, using one simulated dataset from the sample-size calculation. The y-axis shows the predicted number of seizures over 28 days, and the x-axis shows time in months. The number of seizures at time zero varies across patients, as illustrated by the random intercepts. The number of seizures decreases when time on treatment is no longer zero.
